# The Impact of Food Histamine Intake on Asthma Activity: A Pilot Study

**DOI:** 10.3390/nu12113402

**Published:** 2020-11-05

**Authors:** Emilia Vassilopoulou, George N. Konstantinou, Anastasia Dimitriou, Yannis Manios, Lemonica Koumbi, Nikolaos G. Papadopoulos

**Affiliations:** 1 Department of Nutritional Sciences and Dietetics, International Hellenic University, 57400 Thessaloniki, Greece; lemonica.koumbi@gmail.com; 2 Department of Allergy and Clinical Immunology, 424 General Military Training Hospital, 57400 Thessaloniki, Greece; gnkonstantinou@gmail.com; 3 Allergy Department, 2nd Pediatric Clinic, University of Athens, 11528 Athens, Greece; drdimiana@gmail.com (A.D.); ngpallergy@gmail.com (N.G.P.); 4 Department of Nutrition and Dietetics, Harokopio University of Athens, 70 El. Venizelou Avenue, 17671 Kallithea, Greece; manios@hua.gr

**Keywords:** asthma, nutritional intervention, food histamine, Mediterranean diet, Western diet, antioxidants, anti-inflammatory diet

## Abstract

Asthma is a complex chronic inflammatory disorder. Diet’s impact on asthma symptoms is controversial. The objective of this pilot crossover, randomized, two-period study was to examine the effect of dietary histamine intake on asthma symptoms in twenty-one children with mild intermittent asthma. Children were randomly assigned to either a high- or low-histamine diet, based on the Mediterranean pattern, for 4 weeks. After a 2-week washout period, patients crossed to the alternative diet for 4 additional weeks. Asthma symptoms were assessed at baseline and after the completion of each diet period by a clinician. Daily symptoms and peak flow were recorded throughout the intervention. Adherence to the dietary intervention was assessed via analysis of four random 24-h recalls, for each intervention period. Eighteen children completed the study. Significantly higher mean air flow obstruction was recorded and a trend for prolonged and more severe symptoms was observed during the high-histamine period. Diet may have an active and direct impact on asthma symptoms. Food choice is affected and/or may affect symptoms in children with mild asthma. Diet intervention is promising yet challenging, for asthma control.

## 1. Introduction

Asthma is a common chronic disorder of the airways, considered nowadays more as an umbrella term to describe a constellation of clinical symptoms, including wheeze, breathlessness, chest tightness, and cough, with various underlying pathophysiologies [1]. Both genetic predisposition and exposure to environmental factors, such as allergens or irritants promoting airway inflammation, are involved [2].

Evidence shows that diet plays a significant role in the development and improvement of asthma control. Mediterranean diet, characterized by high consumption of vegetables, fruits, whole wheat products and fish, has been demonstrated to be beneficial on controlling asthma symptoms [3,4]. In contrast, “Western” diets, containing high amounts of processed food, saturated fat, sugar and salt, have been associated with increased asthma activity [5,6]. Other studies, however, have reached conflicting results on the effect of diet in asthma [7,8].

A number of food ingredients, including histamine, are known to have a pharmacological action and have been suggested to affect the clinical course of asthma, but their exact impact remains unclear [9,10]. Histamine is contained in food mainly in alcoholic beverages such as red wine or highly processed or spoiled food such as cheese and canned fish, while it can also be found in other food sources after the decarboxylation of the amino acid histidine. Histamine’s content in food varies considerably even within the same food product and is highly affected by the product’s maturity, storage time and processing [11]. It can be metabolized either by oxidative deamination by diamine oxidase (DAO) or by ring methylation by histamine-N-methyltransferase (HNMT). Notably, the last is regarded as the key enzyme for histamine degradation in the bronchial epithelium [10].

The ingestion of histamine-rich foods results in histamine release and blocks the DAO enzyme that is involved in histamine metabolism [11]. A disequelibrium of accumulated histamine and the incapacity of histamine degradation leads to histamine intolerance [11]. The symptoms of this non-allergic adverse reaction involve several organs, such as skin and gastrointestinal system, but also the cardiovascular and respiratory systems, depending on the plasma histamine levels. Specifically, a slight increase in plasma histamine concentrations (i.e., 1–2 ng/mL) has been shown to exert gastrointestinal symptoms or tachycardia, while a 10-fold higher concentration (i.e., 7–12 ng/mL) can lead to a bronchospasm [11]. As recently reported, food histamine intolerance manifests with the appearance of symptoms such as wheeze and asthma [11] and the proposed mechanism for asthma exacerbations is the reduction of HNMT activity [10].

Undisputedly, cellular histamine is involved in asthma pathophysiology. Apart from the mast cells and basophils, there is evidence that cellular histamine is also produced significantly by some gut microbiome phyla in non-obese asthmatic patients [12,13,14]. Diet has a predominant role in the gut microbiota composition and its metabolic activity, but the precise role of histamine-producing bacteria and of the ingested histamine in the disease course is yet to be discovered.

The present pilot study aims to investigate the potential of controlling dietary histamine intake and whether this can interfere with the development of respiratory symptoms in children with mild asthma.

## 2. Materials and Methods

### 2.1. Patients

Twenty-one children (12 boys), diagnosed and followed up in an outpatient allergy unit of a tertiary pediatric hospital in Athens, with mild intermittent asthma (Global Initiative for Asthma (GINA) Stage 1) [15] and documented forced expiratory volume (FEV1) reversibility, were enrolled in the study.

Exclusion criteria included failure to comply with study procedures, presence of other chronic or acute diseases (except rhinitis, allergic conjunctivitis or atopic dermatitis), systematic treatment for diseases other than asthma, and food allergy history. Children and their parents were informed about the study’s protocol and procedures and signed consent was obtained. The parents/caregivers gave their signed informed consent for all the children included in the study before their participation. The study was conducted in accordance with the Declaration of Helsinki, and the protocol was approved by the Scientifical Council and Bioethics committee of the Children Hospital Panagiotis & Aglaia Kyriakou Scientific (Project identification code: issue 3; 130406).

### 2.2. Study Design

A two-period, two-intervention, randomized crossover design was used that, depending on the histamine content, included two types of diet, a low-histamine (LH) and a high-histamine (HH) diet (Table 1). Participants were randomly assigned following simple randomization procedures (computer-generated random numbers) to start with one out of the two proposed diet patterns for eight weeks and continued with the other one for another eight weeks, with a two-week washout period in between. During this time, the volunteers could consume their typical diet without any restriction.

Foods from all groups (dairy, fruits, vegetables, starch, meat, fat) were ranked according to their histamine content into low or high histamine [9,16,17,18,19]. A food list (Table 1), together with eight model diet plans (1800–2000 Kcal; 50% carbohydrates–15% protein–35% fat of which <10 saturated fat) based on the Mediterranean diet prototype, i.e., with a high content of olive oil, whole wheat products, fish, legumes, fruit and vegetables [3], one for each week of the 8-week intervention period, were prepared (example on Table 2). Considering the high variability on histamine content within the same product depending on maturity, storage and processing, a specialized dietitian encouraged patients and their parents to strictly select food products from the LH or HH food list when they were required to make modifications in the respective daily diet plan. Especially for the LH period they were instructed to select fresh not mature of the respective listed fruit and vegetables, to consume fresh meat and fish and to completely avoid frozen and canned products, as well as ready-prepared or packaged food that contain preservatives. Therefore, food recipes for preparing homemade food like juices, jams, burgers, sauces like tomato sauce, lemonade, bread, salads, cake were provided. Hand measures were used to instruct the food portion size measures [20], but each patient decided his portion size ad libitum to avoid weight changes.

Before randomization (t0), participants were evaluated by a clinician to confirm mild intermittent asthma according to GINA [15] and controlled with low inhaled corticosteroids (fluticasone propionate pMDI 100 μg bid). Basic anthropometric measurements (weight and height) were also recorded.

The best out of three morning and evening Peak Expiratory Flow Rate (PEFR) were measured with a flow meter and recorded on diary cards. A reduction of at least 15% reduction in airflow was considered as an asthma episode [21]. Daily symptom scores [22] were documented on diary cards throughout the study. Upper airway symptoms included blocked/stuffy nose, runny nose, sneezing/itchy nose, itchy/sore/watery eyes, hoarse voice, and sore throat. Lower airway symptoms included cough and wheezing/noisy breathing during the day, the night and during exercise. Each symptom was scored from 0 (no symptom) to 3 (severely troublesome). A sum of at least 3 score units for either the upper or the lower respiratory symptomatology was considered as a “*higher score*” day. Symptoms were quantitatively associated and compared between the different dietary intervention periods. The recorded symptom scores (upper, lower or total) were evaluated and analyzed.

After the end of the first (t1) and second (t2) intervention period, asthma symptom records were re-assessed on site, and weights were measured. At t1, guidance for the cross-arm diet plan was provided, for patients to proceed to the second intervention stage after the two weeks washout period. During the intervention periods, eight 24 h recalls were recorded randomly by phone during each diet intervention, in order to assess compliance and provide assistance for better diet implementation. Histidine intake, which is metabolized to histamine, together with the overall energy, macronutrients and micronutrients were further determined via the 24-h diet recalls with the Food-Processor Nutrition Analysis Software [23]. A post-hoc analysis of food choices during the intervention period was performed to evaluate preference changes depending on the provided food lists and symptom fluctuation. Adherence to the Mediterranean diet was explored via the KIDMED questionnaire [24].

**Figure 1:**
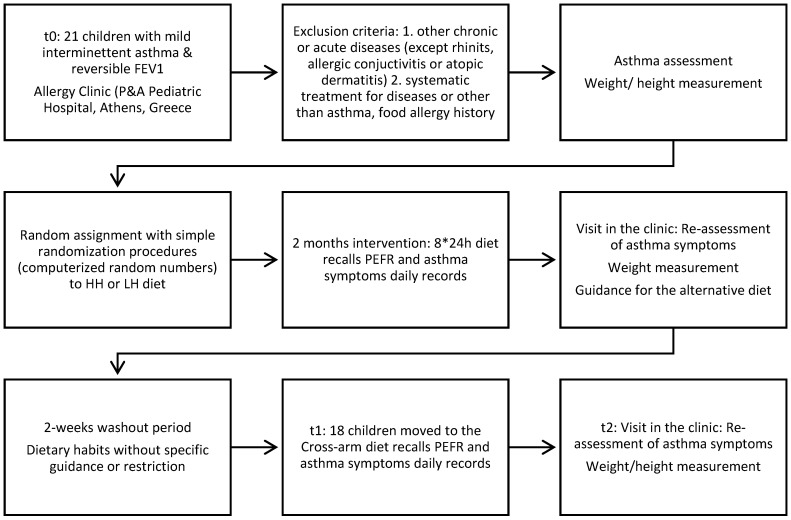
Participant flowchart.

Participant’s flowchart with a description of the patients’ inclusion criteria and study’s design is shown in Figure 1.

### 2.3. Statistical Analysis

The distributions of all recorded parameters were assessed with the Shapiro–Wilk test. Descriptive statistics are presented as median (inter-quartile range) for non-normally distributed variables and means ± standard deviation for normally distributed parameters. The Wilcoxon rank-sum test and Kruskal–Wallis tests were used to compare continuous variables and the Pearson’s X^2^ test to compare categorical variables among studied groups. The Wilcoxon matched-pairs rank-sum test was used to compare continuous variables within the same individual between the multiple measurements. All reported *p*-values are based on 2-sided tests and compared with a significance level of 5%. Stata 9.1 for Windows (Stata Corp LP, College Station, TX, USA) was used for all statistical calculations and plots.

## 3. Results

Eighteen children (10 boys) (mean age 11.5 ± 3.1 years) completed the study successfully. Mean weight at entry point was 47.3 kg (±11.9), mean height was 1.37 m (±9.3) and according to the respective weight to age and height to age growth charts they were within the normal range. No significant weight changes were observed between the different intervention periods (*p* = 0.5).

Mean % predicted FEV1 was 92.6 (±8.6) and no loss of asthma control was recorded during the study period. Nevertheless, the mean PEFR during the LH period was 400 L/min (±55.9) and significantly higher (*p* = 0.0006) than the respective mean PEFR during the HH period 388 L/min (±59). Both upper and lower respiratory symptom scores were lower during the LH period, but the relevant statistical comparisons were not significant. In addition, the median number of symptom-free days had a two-fold increase during the LH diet period as compared to the HH (5.5 days vs. 9.5), but the differences were non-significant (*p*-value = 0.753) (Table 3).

Patients’ dietary habits during the last year before the intervention were characterized by a high consumption of red meat, processed foods, saturated fat, sodium and sugar and low consumption of fruit, vegetables, legumes and fish. In line with this observation, adhesion to the Mediterranean diet was found low with a need to be improved (mean KIDMED score = 4 ± 2).

Upon intervention, adherence to the diet protocol was assessed via analysis of the 24-h diet recalls, in terms of types of foods consumed (Table 4) as well as macronutrient and micronutrient intake. Histidine intake was found to be higher in the HH diet (Table 5). As anticipated, the food choices varied among the LH and HH periods. During the HH diet, milk, junk food, juices and vegetables intake was higher at symptom-free days than during the LH diet. At higher symptom score days, the intake of oils and butter, fish, chocolate, egg and junk food were higher in the HH diet than in LH. Fish consumption was generally low but increased during days with higher symptom scores independently of the diet assigned, possibly due to caregivers’ belief that is a “healthy” choice to relieve symptoms.

Moreover, symptom fluctuation affected food choices within the same intervention period. In the symptom-free days during the HH diet, children consumed more starchy foods, olive oil, chocolate, bacon, ham or sausages and junk food, than in the days with higher symptoms when they consumed more fish and butter. Respectively, in the LH symptom-free days, individuals chose more starchy products, juices from permitted fruit and permitted ham. On the contrary, at higher symptom days during the LH diet, they consumed more olive oil and fresh fish.

In terms of micronutrients, inadequate intake of vitamin D, potassium, and manganese was noted at all stages of intervention. Marginally lower intake of calcium and folate compared to the Dietary Reference Intakes (DRIs) [25] were noted during LH. Furthermore, although zinc’s intake was sufficient at all stages, it was double during the HH stage (Table 5).

## 4. Discussion

Childhood asthma is a complex disease and evidence indicate that diet plays a role on its clinical outcome. This randomized crossover dietary intervention aimed to evaluate the effects of dietary histamine in children with mild asthma. Our results clearly show a significantly higher airflow obstruction during the high-histamine period, whereas a trend towards less symptoms and more symptom-free days during the low histamine intake diet periods was observed.

Histamine is a potent inflammatory mediator and is associated with deleterious inflammation in asthmatic patients, triggering airway hyper-responsiveness and remodelling. The microbial community in the intestine is shaped by the diet and supplies the host with a number of metabolites, including short-chain fatty acids and histamine. Histamine-secreting bacteria are found at a higher frequency in faecal samples from asthmatic patients. The possible contribution to their atopic phenotype is a matter of further investigation, through larger-scale studies on the microbial diversity of asthmatics, especially because it is impossible to distinguish the microbial-derived histamine from the human-produced form [14].

Moreover, food histamine has been demonstrated to be a trigger for worsening asthma symptoms [10]. In line with this, we found higher intake of histamine in the form of histidine in the HH period to be associated with higher airflow obstruction and symptom exacerbation. Histamine intolerance in asthmatics could lead to dyspnea or dysphonia provoked after high histamine ingestion [10]. Notably, histidine is not the only source of histamine from food but is also an autochthonous ingredient in some foods produced by different cooking preparations [17]. Therefore, this finding can be partly accepted as a measure of compliance and as a separate notion.

As the role of diet on asthma symptoms is complex, dietary plans implemented herein were designed under Mediterranean-style eating pattern. This was decided in order to control at most the overall nutrient intake and for the benefit of the patients of the study since it is generally accepted that the specific diet pattern reduces inflammation [7]. Indeed, the analysis of the diet-recalls revealed a desirable high consumption of olive oil (MUFA), as the main source of fat at all stages of the intervention.

The anti-inflammatory n-3 PUFA [26,27,28,29] intake though, originating from fish oil, was generally low. Interestingly, when children in both intervention phases developed symptoms, they increased fish consumption. It can be assumed that caregivers considered fish as a “healthy” choice to relieve symptoms. Similarly, our cases presented a deficient vitamin D intake at all times, which has been previously correlated to pulmonary dysfunction in childhood [30], but consumed more butter during the symptomatic periods, which is an external source of vitamin D. Adequate vitamin D intake has been suggested to reduce the number of exacerbations, the requirement of steroids and emergency visits in asthmatic school-aged children. Although no safe conclusions can be produced for the role of vitamin D in this intervention, further evaluations are needed about its beneficial role in asthma, as results so far are unclear [31,32].

Saturated fat intake, originating from milk, egg, fried food, and meat products, was generally high at all intervention stages. Past habits of our participants were closer to the Western diet, with a high intake of SF, refined grains and sugar. Patients were advised at the beginning of the intervention to follow the provided model diets but had the liberty to make changes with foods from the respective provided food list, according to their personal preferences and established dietary habits. As such, they often chose processed foods with higher saturated fat and sugar content. Nevertheless, and although saturated fat is associated with the induction of proinflammatory mediators [33], SF intake was equal in both intervention stages, so no further estimation of its influence can be made.

On the other hand, during the LH period, beta-carotene intake was superior. Beta-carotene is an important antioxidant that has been directly involved in asthma pathogenesis [34]. This positive effect could have added to the shorter and milder periods of symptoms encountered during the low-histamine diet. During the same intervention period, zinc intake was recorded within the recommended DRI’s but lower in comparison to the HH period. Zinc has been previously suggested to increase antioxidant function and reduce asthma exacerbations [35]. Nevertheless, these data cannot be better evaluated in the current study’s context.

As elucidated from our results, a diet intervention aiming to control a single nutrient’s intake is really challenging unless, as elsewhere shown, this is based on a food supplement [36] or ready-prepared meals to control overall intake [37,38]. Both these strategies, though, do not reflect the ability of patients to adapt to the proposed changes of eating habits, nor provide the long-term effect of dietary changes [39]. Irrespective of this, such change is generally difficult to adopt and maintain unless proper therapeutic techniques from medical nutrition and behavioral therapy are incorporated [40].

The present study suggests that food histamine has a significant impact on asthma outcome in children who show a trend for a more “healthy diet” during symptom deterioration periods. Our findings show a significance in mean airflow obstruction and a trend for more symptoms with higher duration during the high-histamine period. As the last did not reach significance, probably due to the small sample of participants, the short intervention period and the mild symptomatology of the selected patients, we could aim for future studies with longer intervention periods, a bigger number of participants or participants with more persistent or severe asthma to confirm this effect.

## 5. Conclusions

Eating behaviors change in asthmatics during days with symptoms, as also shown here. Histamine intake, along with other dietary factors, influences asthma. In order for the actual effect on the clinical outcomes to emerge, further exploration with long-term dietary interventions are needed, keeping in mind that a diet intervention is promising yet seriously challenging.

## Figures and Tables

**Table 1:** Food ranking in low and high histamine content.

Food Group/Type of Diet	Low Histamine	High Histamine
Dairy products	Fresh pasteurised plain milk without additives (cow’s or goat’s), butter, plain yogurt, ricotta cheese, cottage cheese, cream cheese	All mature cheese, e.g., brie, camembert, cedar, Cheshire, Danish blue, edam, emmental, Gloucester, gouda, mozzarella, parmesan, Roquefort, yogurt with additives such as fruits, cereal, cacao milk, chocolate milk, plain whole fat long life milk
Fruit	Fresh not very mature apple, apricot, peach, pear, melon	Mature dates, kiwi, orange, papaya, tangerine, passion fruit, avocado, banana, fig, grape, lemon, pineapple, plum, berry, strawberry
Vegetables	Asparagus, cabbage, lettuce, green beans, onion, pepper, radish, turnip	Broccoli, cauliflower, eggplant, mushroom, tomato, sauerkraut, spinach, cucumber
Legumes	Peas, lentils, chickpeas	Beans, soybeans
Starchy products/grains	Bread with natural yeast without preservatives, oat cereals, pure corn flakes, potato, sweet potato, rice, spaghetti, any type of pasta excluded pasta stuffed with cheese, mince meat, cold-cuts, (e.g., tortellini) and colored pasta (with spinach, carrot, etc.	Bread with preservatives, long life bread, stuffed pasta with cheese, mince meat, cold cuts, colored pasta, cereals with chocolate, cereals with fruits, cereals with nuts, ready-made meals (frozen)
Nuts	Chestnut, sunflower seeds, pine nuts, pistachios, almonds, coconuts, currants, red currants, cashew nut, macadamia nut	Hazelnuts, brazil nuts, walnuts, pecan, peanuts, peanut butter
Spices	Herbs, spices except for anise, red pepper, curry (ready-made spicy products not allowed)	Malt vinegar, meat products, soy sauce, vinegar, Worcestershire sauce, yeast products
Meat/poultry/fish	Beef, chicken without skin, turkey without skin, fresh fish, lamb, rabbit, veal, cooked eggs, boiled unsalted turkey	Meat, fish and poultry older than two days, any frozen meat, any form of raw or smoked meat or fish, anchovies, beef liver, pies and pasta with meat, fish nuggets, salami, sausages, chicken livers, canned tuna, canned salmon
Fats/oils	All vegetable oils, homemade sauces prepared with permitted ingredients	Ready-made sauces, olives, oils with preservatives and colorants, olives
Beverages	Homemade lemonade	Tea, decaffeinated tea, ready-made juices, chocolate drinks, cacao-drink, soft drinks including cola-type, tomato juice, vegetable juices
Sweets/sweeteners	Sugar, homemade jams, homemade sweets with permitted ingredients	Cocoa, milk chocolate, black chocolate, white chocolate

**Table 2:** Model weekly diet plan provided to the patients for the low-histamine (LH) and high-histamine (HH) period of the dietary intervention.

*Low Histamine* *							
Breakfast	1 cup of fresh whole fat plain milk+ 2 slices of whole wheat bread made with fresh yeast+ 1tsp butter+ 1tsp honey	1 cup of fresh whole fat milk+ 1slice of whole wheat bread made with fresh yeast+ 1tsp homemade peach jam	1 cup of fresh whole fat plain milk+ 3 whole wheat bread rusks	1 cup of fresh whole fat plain milk+ 1 whole wheat bread+ 1tsp honey	200gr whole fat Greek yogurt+ 1 pear	1 cup of fresh whole fat plain milk+ 2 slices of whole wheat bread made with fresh yeast+ 1tsp butter+ 1tsp honey	1 cup of fresh whole fat plain milk+ 2 slices of whole wheat bread made with fresh yeast+ 1tsp butter+ 1tsp honey
Snack	1 cup melon	1 cup of fresh apple-pear juice	1 apple	1 pear	1 cup of fresh juice from apple-pear	1 apple	1 cup of fresh apple juice
Lunch	1 portion green fresh beans+ 1 slice of whole wheat bread+ potatoes	1 portion stuffed zucchini or peppers with rice and beef or turkey mince meat+ 1slice of whole-wheat bread	1 portion of boiled fresh fish+ 1 boiled potato+ boiled carrots, zucchini	1 portion ratatouille vegetables (zucchini, carrots, peppers, onion, olive oil, potatoes)+ 1 slice whole wheat bread	1 portion of pasta with turkey/chicken/beef mince meat+ boiled zucchini+ olive oil	4–5 fried meatballs with chicken mince meat+ 15–20 slices of fried potatoes+ lettuce salad	1 portion leek with rice, lemon and olive oil+ 1 slice whole wheat bread+ cottage or cream cheese
Snack	Oven-baked apple with egg and breadcrumbs, sugar and cinnamon	1 milkshake with fresh whole fat plain milk, vanilla flavour, honey	Homemade popcorn with olive oil or butter	1 slice of homemade vanilla cake	1 homemade rice pudding	1 full-fat yogurt+ 1tsp honey	1 apple milk shake: full-fat milk, apple, cinnamon, honey
Dinner	Homemade Caesars Chicken salad with lettuce, boiled corn, cucumber, olive oil	1 homemade chicken burger with whole wheat bread, lettuce	1 homemade sandwich with lettuce, cottage cheese, boiled turkey ham, lettuce	1 homemade souvlaki with chicken meat and whole wheat pita bread, fried potatoes	Egg salad: egg, tomato, lettuce, olive oil	Lettuce salad with baked lamb meat	Baked chicken meat with cucumber-lettuce salad and olive oil
*High Histamine* *							
Breakfast	1 cup of whole fat long life milk+ 2whole wheat homemade chocolate biscuits	1 cup of whole fat long life milk+ 1slice of whole wheat bread+ 1tsp Nutella	1 cup of whole fat long life chocolate milk+ 2 homemade vanilla biscuits	1 cup of whole fat long life milk+ 1 whole wheat bread+ 1tsp honey	1 fruit stirred yogurt with strawberries	1 cup of whole fat long life milk+ ½ cup chocolate cereals	1 cup of whole fat long life milk+ 2 slices of toasted bread+ 2tsp strawberry jam
Snack	1 cup of fresh orange juice	1 cup of packed mixed fruit juice	1 banana	1 cup of packed anana juice	1 banana	1 kiwi fruit	1 cup of packed mixed fruit juice
Lunch	1 portion of lentils+ mature yellow cheese (Cretan graviera)+ 1slice of wholewheat bread	1 portion stuffed tomatoes with rice and frozen mince-meat+ 1slice of wholewheat bread+ feta cheese	1 portion of frozen fish sticks+ 1boiled potato+ cauliflower or broccoli	1 portion “tourlou” vegetables (aubergine, carrots, cucumber, tomato, olive oil, potatoes)+ 1 slice whole wheat bread+ feta cheese	1 portion pasta with tomato sauce and mushrooms+ parmesan cheese+ Tomato salad	4-5 fried meatballs from frozen minced-meat+ 15-20 slices of fried potatoes+ broccoli or cauliflower	1 portion spinach with rice and tomato+ feta cheese+ 1 slice of whole wheat bread
Snack	1 yogurt with chocolate slices	Homemade Banana milkshake (banana, whole milk, honey)	Fruit salad: grapes, orange, banana	1 cup strawberries or grapes	chocolate ice cream	50gr milk or back chocolate	Lemon or strawberry Sorbet
Dinner	1 piece of ham and cheese pie	1 burger with pork mince neat_ tomato salad	1 hot dog: whole wheat bread, 1 sausage stuffed with cheese, 1tsp mustard, 1tsp ketchup	1 souvlaki with pork meat and whole wheat pita bread, fried potatoes, tomato	Chef salad: Roquefort cheese, salami, cedar, boiled egg, tomato, lettuce, olive il	1 slice of spinach pie	Baked pork meat with Greek salad

* All meals and salads prepared with fresh olive oil.

**Table 3:** Comparisons between the median number of days with symptoms and median symptom score during the two intervention periods.

		Type of Diet		
		Low Histamine	High Histamine	*p*-Values **
Symptoms duration in days		5.5 (8)	9.5 (4)	0.753
Respiratory Symptoms	Upper *	4.67 (11.5)	12.5 (5.83)	0.780
	Lower *	4.25 (13.1)	14 (12.45)	0.727
	Total *	11.33 (53.5)	30.17 (21.42)	0.889

Data are presented as median (interquartile range). * units in symptom score scale; ** comparisons between different types of diets (Wilcoxon matched-pairs sign test).

**Table 4:** Changes in the frequency of consumption of different foods (food choices) according to symptom fluctuation during and among the two intervention periods.

	LH Period			HH Period			HH vs. LH	
	Remission	Episode		Remission	Episode		Remission	Episode
			*p*-Value ^1^			*p*-Value ^1^	*p*-Value ^2^	*p*-Value ^3^
Milk	1.74 (±1.38)	2.69 (±1.21)	0.064	2.69 (±1.98)	3.77 (±1.35)	0.133	0.004	0.107
Starchy food	3.65 (2.94)	6.04 (2.72)	0.021	4.61 (3.28)	6.27 (±1.85)	0.046	0.972	0.141
Meat	4.01 (±2.91)	5.19 (±2.32)	0.382	3.25 (±2.05)	4.4 (±1.78)	0.133	0.196	0.207
Olive oil	3.24 (±3.12)	0.26 (±0.44)	0.014	0.28 (±0.44)	4.23 (±3.16)	0.002	0.002	0.007
Butter	0.318 (±0.83)	0.05 (±0.14)	0.299	2.56 (±2.62)	0.46 (±0.5)	0.043	0.014	0.007
Fish	0.51 (±0.89)	0.75 (±0.59)	0.184	3.7 (±2.93)	0.29 (±0.47)	0.003	0.059	0.003
Chocolate	0.268 (±0.7)	0.28 (±0.45)	0.657	0.9 (±0.74)	3.33 (±3.06)	0.007	0.002	0.015
Egg	0.05 (±0.15)	0.04 (±0.08)	0.809	0.89 (±1.47)	0.18 (±0.34)	0.053	0.027	0.006
Sweats	0.74 (±0.8)	1.12 (±1.1)	0.328	1.55 (±1.34)	1.46 (±1.08)	0.861	0.463	0.123
Nuts	0.67 (±1.3)	0.4 (±0.42)	0.528	0.61 (±1.39)	0.09 (±0.19)	0.426	0.035	0.514
Bacon or sausages or ham	0.11 (±0.18)	5.08 (±3.21)	0.002	0.31 (±1.11)	0.71 (±0.46)	0.030	0.002	0.242
Junk food	1.01 (±1.27)	0.89 (±1.15)	0.889	0.12 (±0.3)	2.03 (±1.41)	0.002	0.055	0.017
Fried oil	0.79 (±1.25)	0.14 (±0.13)	0.726	1.23 (±1.59)	1.26 (±0.94)	0.917	0.003	0.105
Juices	0	0.8 (0.76)	0.004	0.15 (0.56)	0.12 (0.2)	0.155	0.016	0.317
Fruit	0.29M (±0.37)	0.82 (±0.61)	0.546	1.27 (±2.35)	0.59 (±0.49)	0.726	0.221	0.302
Vegetables	0.66 (±1.02)	0.1 (±0.23)	0.052	0.4 (±0.51)	0.95 (±0.79)	0.028	0.002	0.806

Changes in food choices are presented in mean (±standard deviation). Comparisons (Wilcoxon matched-pairs rank-sum test) of food choices: ^1^ during episodes vs. remission periods within the same intervention period; ^2^ during remission between the two intervention periods; ^3^ during episodes between the two intervention periods.

**Table 5:** Comparison of micronutrient intake (mean values) with Dietary Reference Intake (DRI).

Type of Diet	High Histamine	Low Histamine	DRI
Sodium (mg) **	3200	1808	1500
Potassium (mg)	2922	2557	4500
Calcium (mg)	1365	807	1300
Iron (mg)	14.5	12	8
Phosphorus (mg)	1603	1208	1250
Magnesium (mg)	280	240	240
Zinc (mg) **	22	8.9	8
Copper (mg)	1.3	1	0.7
Manganese (mg)	1.6	1.7	1.9
Selenium (mg)	0.13	0.1	0.04
Fluoride (µg)	223	300	200
Chromium (mg)	0.03	0.04	0.025
Molybdenum (µg)	43	49	34
Histidine (mg) **	2400	1800	NA
Vitamin A (RE)	637	627	600
Beta-carotene (ug) **	217	494	NA
Vitamin C (mg)	54	46	45
Vitamin D (ug)	4.5	4.46	15
Vitamin E (IU)	11.6	10.4	11
Thiamin (mg)	2	1.26	0.9
Riboflavin (mg)	2.4	1.67	0.9
Niacin (mg)	21.5	20.4	12
Vit B6 (mg)	1.65	1.5	1
Vit B6 (mg)	1.65	1.5	1
Folate (µg)	336	253	300
Vit B12 (µg)	4.98	3.8	1.8
Biotin (µg)	21.5	20.6	20
Pantothenic Acid (mg)	4.4	4	4
Vitamin K (µg)	30	40	60

Deficient intake in specific nutrients is marked in bold letters. ** statistically significant difference (*p* = 0.05) for specific nutrient’s intake among the two types of diet. NA: non- applicable.
